# From fragmentation to resolution: high-fidelity genome assembly of *Zancudomyces culisetae* through comparative insights from PacBio, Nanopore, and Illumina sequencing

**DOI:** 10.1093/g3journal/jkaf204

**Published:** 2025-09-01

**Authors:** Huimei Yang, Yan Wang

**Affiliations:** Department of Ecology and Evolutionary Biology, University of Toronto, Toronto, Ontario M5S 3B2, Canada; Department of Ecology and Evolutionary Biology, University of Toronto, Toronto, Ontario M5S 3B2, Canada; Department of Biological Sciences, University of Toronto Scarborough, Toronto, Ontario M1C 1A4, Canada

**Keywords:** Illumina, Nanopore, PacBio-CLR, PacBio-HiFi, Zoopagomycota, mosquito, gut fungi, symbiont, genome assembly

## Abstract

*Zancudomyces culisetae* is an obligate symbiotic fungus inhabiting the digestive tracts of aquatic insect larvae, including black flies, midges, and mosquitoes. With a global distribution and high prevalence in disease-transmitting insects, *Z. culisetae* serves as a model for studying insect gut fungi. A previous draft genome assembly using Illumina short reads provided insights into its genome composition, such as a low GC ratio and evidence of horizontal gene transfer. However, its fragmented nature has limited deeper exploration of the evolutionary mechanisms shaping these gut symbionts. To address this gap, we generated a wealth of genomic resources for *Z. culisetae* using multiple sequencing platforms, including Illumina, Oxford Nanopore, PacBio-CLR (Complete Long Reads), and PacBio-HiFi (High Fidelity). This also provides an opportunity to compare these popular sequencing methods to suggest the optimal approach for fungal genome assembly. Our results suggest that PacBio-HiFi produced the most complete assembly, yielding a 27.8 Mb genome size with 26 contigs, representing the highest-quality genome of insect gut fungi to date. Additionally, we generated transcriptomic data to support genome annotation, identifying 8,484 protein-coding genes. Despite the improved genome quality, *Z. culisetae* lacks ∼20% of Benchmarking Universal Single-Copy Orthologue commonly found in fungi, reflecting adaptations to its obligate symbiotic lifestyle. This study not only provides valuable genomic resources for insect gut fungal research but also evaluates the strengths and limitations of current genome sequencing and assembly approaches, offering best practices for fungal genome analysis and genetic research.

## Introduction

*Zancudomyces culisetae* (formerly known as *Smittium culisetae*) is a well-studied insect gut-dwelling fungus, belonging to an important lineage of early-diverging fungi (Harpellales, Zoopagomycota) (White 2006; Wang et al. 2013; Spatafora et al. 2016). *Zancudomyces culisetae* has been frequently reported worldwide, primarily colonizing the hindgut of disease-vector insect larvae, including black flies, mosquitoes, and midges (Lichtwardt 1986, 1999; Valle and Santamaria 2004; White et al. 2006; Chaudhary et al. 2023). These fungi have maintained an obligate symbiotic relationship with dipteran hosts for ∼270 million years, coinciding with the evolution of complete metamorphosis in insects (Wang et al. 2019). This ancient symbiosis offers a unique model for studying microbial adaptation to extreme environments, such as insect guts, and the long-term co-evolution between fungi and one of Earth's most ecologically dominant animal groups.

The first *Z. culisetae* culture was isolated from a fourth-instar larva of *Culiseta incidens* and successfully grown on SNB-9 medium and Difco brain-heart infusion agar (Clark et al. 1963). Using similar techniques, additional axenic cultures were established from various locations (Lichtwardt 1964; Lichtwardt and Williams 1990; Strongman and White 2008). These cultures enabled extensive research into the biology and symbiotic interactions of insect gut-dwelling fungi, including their lifecycle, nutritional requirements, and host distribution (Lichtwardt 1964; Horn and Lichtwardt 1981; Williams 1983; Horn 1989; Sato 1992; McCreadie and Beard 2003; Nelder et al. 2005; Vojvodic and McCreadie 2009). More recently, molecular systematics have supported the independent placement of *Z. culisetae* outside *Smittium*, where it was originally classified (Wang et al. 2013, 2014; Tretter et al. 2014). The successful cultivation of *Z. culisetae* facilitated the whole genome sequencing using Illumina short reads, leading to the discovery of a horizontally transferred polyubiquitin gene from mosquito hosts (Wang, White, Kvist, et al. 2016). Subsequent genomic studies on insect gut fungi have uncovered a noncanonical genetic mechanism in which the UGA stop codon is repurposed to encode the 21st amino acid, selenocysteine, in fungi for the first time (Mariotti et al. 2019). However, the currently available *Z. culisetae* genome assembly (COL-18-3; ARSEF 9012) was generated using Illumina short-read sequencing, resulting in a highly fragmented assembly of 1,954 scaffolds (Wang, White, Kvist, et al. 2016). This fragmentation limits further investigation into critical molecular mechanisms, such as horizontal gene transfer and selenocysteine incorporation via stop codon reassignment. Additionally, no RNA sequencing data are available for accurate gene annotation or expression analysis in *Z. culisetae*.

To address these limitations, we present a reference-quality genome assembly of *Z. culisetae* in this study. We sequenced the HAW-14-8 strain (ARSEF 9014), originally isolated from the hindgut of an *Aedes albopictus* mosquito in Oahu (Hawaii, USA). By integrating cutting-edge sequencing technologies, including PacBio Single-Molecule Real-Time Sequel II platform (for both continuous long reads and circular consensus sequencing reads, also known as High-Fidelity reads), Oxford Nanopore PromethION, and Illumina NovaSeq 6000, we compared the performance of different sequencing platforms and assembly strategies for gut-dwelling fungi. The final reference-quality genome was assembled using PacBio HiFi data, which produced the highest-quality assembly. However, this assembly exhibited relatively low completeness scores when assessed using Benchmarking Universal Single-Copy Orthologue (BUSCO) with the fungi_odb10 dataset. This dataset is widely used across fungal lineages, including Zoopagomycota, where free-living soil saprobes typically show high completeness scores (Chang et al. 2015; Amses et al. 2022; Wang et al. 2023). Notably, this pattern is consistent with previously findings from other sequenced gut-dwelling fungal genomes (Wang, White, and Moncalvo 2016; Wang, White, Kvist, et al. 2016; Wang et al. 2018; Prakash and Wang 2024). Our study provides a high-quality genomic resource that will facilitate further investigations into the evolutionary adaptation, molecular interactions, and functional genomics of insect gut fungi, including possible gene loss events due to symbiotic interactions with mosquito hosts.

## Materials and methods

### Fungal sample preparation and DNA extraction

The *Zancudomyces culisetae* strain HAW-14-8 was originally isolated from *Aedes albopictus* in Oahu, Hawaii, USA (Lichtwardt 1964) and obtained from the USDA-ARS Collection of Entomopathogenic Fungal Cultures (ARSEF 9014). Fungal cultures were grown in Brain Heart Infusion Glucose Tryptone vitamins (BHIGTv) broth at room temperature for 1 week, following the method described by White et al. (2006). Fungal tissues were harvested by filtration through autoclaved Miracloth (MilliporeSigma, Canada) and ground to a fine powder in liquid nitrogen. High molecular weight DNA (>20 kb) was extracted using the MagAttract HMW DNA Kit (QIAGEN, Canada), while mRNA was isolated using the MagMax plant RNA isolation kit (Thermo Fisher). Both DNA and RNA extractions followed the instructions provided by the respective manufacturers.

### Genome sequencing

DNA and RNA libraries (PE150 for 350 bp) were prepared and sequenced on the Illumina NovaSeq 6000 system at the Novogene UC Davis Sequencing Center. Long-read DNA libraries (>20 kb) were prepared and sequenced at the Centre for Applied Genomics, Hospital for Sick Children (Toronto, Canada) using both the ONT PromethION (FLO-PRO002 R9.4 flow cell with SQK-LSK110 ligation sequencing kit) and PacBio Sequel II platforms (Express TPK 2.0 + Sequencing primer v4) separately.

### Genome assembly

Illumina short reads were adapter-trimmed using Trimmomatic v0.36 (Bolger et al. 2014) and quality-checked using FASTQC v0.11.9 before being assembled into contigs using SPAdes v3.13.1 with default k-mer setting (i.e. 21, 33, 55, and 77) for 150 bp read length. Raw electrical signal FAST5 files from the ONT PromethION platform were base-called using Guppy v5.0.12 (https://community.nanoporetech.com), and the resulting reads were trimmed and assembled into contigs using Canu v2.2 pipeline (Zimin et al. 2017). The PacBio CLR raw reads were assembled using Flye (Kolmogorov et al. 2019), while PacBio HiFi raw data were adapter-trimmed with HiFiAdapterFilt v2.0.0 and assembled into contigs using HiFiASM v0.16.1-r375 (Cheng et al. 2021; Sim et al. 2022). FinisherSC was applied to improve the assembly by refining the HiFiASM primary assembly using the trimmed long-read data (Lam et al. 2015). For hybrid assemblies, SPAdes v3.13.1 was used to integrate Illumina short-reads with long-reads from PacBio CLR and/or Oxford Nanopore, leveraging long-read data for gap closure and repeat resolution (Antipov et al. 2016). The genome size was also estimated using Jellyfish v2.3.0 and GenomeScope v2.0, based on Illumina short-read data (Marçais and Kingsford 2011; Ranallo-Benavidez et al. 2020).

### Assessment and comparative analysis of genome assemblies

Genome assembly completeness was assessed using BUSCO v5.1.2 with the fungi_odb10 database (Manni et al. 2021). Assembly statistics were summarized using gfastats v1.3.10 and visualized using the R package “ggplot2” and BlobToolKit v2.6.5 (Wickham 2016; Challis et al. 2020; Formenti et al. 2022). Assemblies generated using different strategies were first reordered against the highest-quality reference (i.e. PacBio-HiFi) using Mauve Contig Mover tool, followed by locally collinear blocks (LCBs) identification via progressiveMauve alignment with default settings (Rissman et al. 2009; Darling et al. 2010). The final assembly (i.e. PacBio-HiFi) was further evaluated using the reference-free tool, Inspector v1.3.1, to assess read-to-contig alignment rate and coverage depth (Chen et al. 2021).

### Mitochondrial genome

To recover the complete mitochondrial genome, we followed the MitoHiFi v2 workflow using the published mitochondrial genome of *Z. culisetae* (GenBank accession: NC_006837.1) as a reference (Seif et al. 2005; Allio et al. 2020; Uliano-Silva et al. 2023). Default parameters were applied, with 2 exceptions: the organism group was set to fungi using the “-a fungi” flag, and the standard genetic code was specified with “-o 1”.

### Genome annotation

RepeatModeler v2.0.2a and Repeatmasker v4.1.0 (https://www.repeatmasker.org) were used to softmask interspersed repeats and low-complexity DNA sequences in the assembly. RNAseq raw data were adapter-trimmed using Trimmomatic v0.36 and mapped to the genome assembly using HISAT2 v2.2.1, followed by processing with SAMtools v1.12. Genome annotation was performed on the softmasked genome assembly using the Braker pipeline v1.9 (parameters −fungus −softmasking 1), which integrates Augustus and GeneMark with RNA evidence for gene prediction. Functional annotation was carried out using InterProScan v5.73-104.0 (Jones et al. 2014). Selenoprofiles v4.4.7 was used to predict selenoprotein families and selenocysteine-related machinery in the *Zancudomyces culisetae* genome and transcriptome (Santesmasses et al. 2018).

## Results and discussion

### Comparative overview of sequencing strategies for *Z. culisetae*

**Fig. 1.**
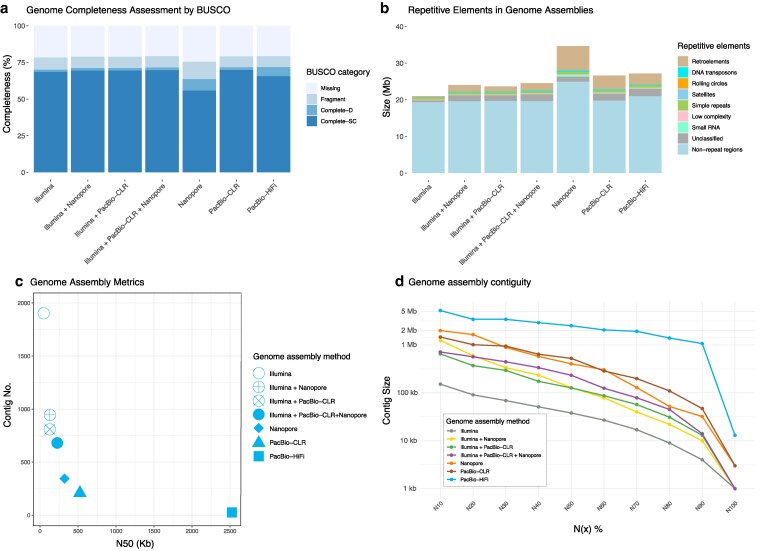
Comparison of genome sequencing and assembly approaches for *Zancudomyces culisetae*. a) Genome assembly completeness assessed using BUSCO for 7 assemblies generated from single-platform (Illumina, Nanopore, PacBio-CLR, or PacBio-HiFi) or hybrid approaches (Illumina + Nanopore, Illumina + PacBio-CLR, and Illumina + PacBio-CLR + Nanopore). Stacked bars represent the proportion of complete (single-copy/duplicated), fragmented, and missing BUSCO genes. b) Repetitive element content annotated with RepeatMasker. Stacked bars show the relative abundance of repeat classes (e.g. Retroelements, DNA transposons, Simple repeats) in each assembly. c) Genome assembly metrics plot comparing N50 (*x* axis) and total contig count (*y* axis). Symbols denote assembly strategies. d) Assembly contiguity profiles plotting contig sizes (*y* axis) against N(x) statistics (*x* axis), where N(x) indicates the length for which the collection of all contigs of that length or longer covering x% of the genome (e.g. N50, N90).

In this study, we compared the performance of Illumina, Oxford Nanopore, PacBio-CLR, and PacBio-HiFi sequencing technologies for de novo genome assembly of *Z. culisetae*. Genome assemblies generated from different sequencing platforms were evaluated for completeness using BUSCO (Fig. 1a). Most assemblies exhibited similar completeness scores, ranging from 70.1% to 71.9% for the Complete category, which includes both Single Copy and Duplicated content. The exception was the assembly generated from Nanopore data alone, which had a lower completeness score of 63.7%. Notably, while Illumina-only and hybrid approaches showed comparable completeness to long-read assemblies, the Nanopore and PacBio-HiFi assemblies displayed a slightly higher proportion of Complete-Duplicated BUSCO content.

Repetitive elements were analyzed and compared across genome assemblies generated using different approaches (Fig. 1b). The total repetitive content varied among assemblies, with long-read assemblies (e.g. Nanopore, PacBio-CLR, and PacBio-HiFi) recovering a larger proportion of repetitive sequences compared with Illumina-only assemblies. Hybrid assembly approaches showed significantly improvement by recovering a greater number of repetitive elements than short-read assembly alone. Interestingly, Nanopore assembly reports the largest number of repetitive elements, contributing to its larger genome size. Among the repeat categories, retroelements constituted the majority of the repetitive content across all assemblies, followed by unclassified repeats, simple repeats, and DNA transposons. Notably, the Nanopore-based assembly recovered the largest region of repetitive elements in nearly every category, including retroelements, DNA transposons, small RNA, simple repeats, and low complexity. Satellites and rolling circles were not consistently recovered across assemblies that the rolling circles were absent in Illumina-only and Nanopore-only assemblies, while satellites were only detected in hybrid assemblies (Illumina + PacBio-CLR and Illumina + PacBio-CLR + Nanopore).

**Fig. 2.**
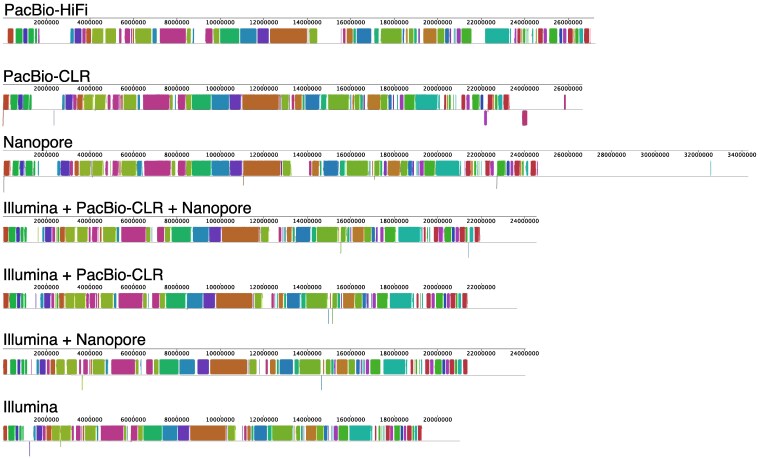
Whole-genome alignment of *Zancudomyces culisetae* assemblies using progressiveMauve. The PacBio-HiFi assembly (top row) serves as the reference for comparison with other assemblies (labeled rows below). The horizontal axis represents the size of each genome assembly. Locally collinear blocks (LCBs) are shown as colored regions, with shared colors indicating homologous sequences across assemblies. Inverted blocks (reverse orientation) appear below the horizontal axis. White regions indicate sequences that did not meet the minimum weight threshold set by the progressiveMauve algorithm.

To evaluate the quality of genome assemblies generated by different sequencing strategies, we compared key assembly metrics, including N50 and contig counts (Fig. 1c), as well as contig length statistics (Fig. 1d). Assemblies produced using long-read technologies (e.g. PacBio-CLR, PacBio-HiFi, and Nanopore) consistently achieved higher N(x) values (e.g. N50 and N90; Fig. 1d) and fewer contig numbers (Fig. 1c), indicating improved assembly continuity. Among all utilized methods in this study, the PacBio-HiFi assembly outperformed others, exhibiting the highest assembly statistics, including N50 (2.5 Mb) and the fewest contigs (26), reflecting superior contiguity and minimal fragmentation (Fig. 1c and d). In contrast, the assembly generated using Illumina-only reads showed the lowest N50 (38 Kb) and the largest number of contigs (1,898), indicating significant fragmentation and large number of gaps remained within the assembly. Hybrid assembly approaches, which combine Illumina short-read data with long-read data for gap closure and repeat resolution, demonstrated improved performance (Fig. 1d). For example, combining Illumina with Nanopore data significantly reduced the contig number (from 1,898 to 953) and increased the N50 score (from 38 Kb to 129 Kb) (Fig. 1c). Similarly, the combination of Illumina and PacBio-CLR data resulted in fewer contigs (from 1,898 to 810) and a higher N50 score (from 38 Kb to 127 Kb). The metrics improved further when using both types of long-read data, yielding 688 contigs and an N50 of 233 Kb. These results highlight that enhanced continuity can be achieved by incorporating long-reads to Illumina short-read assemblies. Overall, assemblies based solely on long-read data generally performed better, with the PacBio-HiFi assembly yielding the best results: a 27.2 Mb genome comprising 26 contigs and an N50 of 2.5 Mb. This closely matches the estimated genome size of 26.2 Mb, based on Jellyfish and GenomeScope analyses. Additional metrics further support the high quality of the PacBio-HiFi assembly, including a 95.9% read-to-contig alignment rate and a mean read coverage depth over 48×. Accordingly, the PacBio-HiFi assembly was used as the reference for whole-genome alignment of *Z. culisetae* assemblies generated from different sequencing platforms and combinations, as visualized with progressiveMauve (Fig. 2). This comparison serves as a technical benchmarking exercise for sequencing the genome of the same fungal strain (HAW-14-8). Most LCBs are recovered across all assemblies; however, the interspersed sequences between LCBs are not consistently present and appear reduced in size, particularly in the Illumina-based assemblies. Notably, the unaligned regions at the far right of each assembly are most extensive in the Nanopore assembly, contributing to its overall larger genome size compared to the other assemblies.

### Recovery of repetitive DNA

Our results show that different sequencing platforms vary in their ability to recover duplicated regions and repetitive elements. Illumina-based assemblies contained less repetitive content (1.7 to 4.9 Mb), with repeat content increasing when long-read data were included (Fig. 1b and Table 1). Notably, the PacBio-HiFi assembly recovered significantly more retroelements (3.03 Mb) compared to the Illumina-only assembly (0.41 Mb; Table 1 and Fig. 1b), consistent with the superior ability of long reads to resolve repetitive sequences, including segmental duplications and transposable elements, which often self-duplicate and generate large, repeat-rich regions (Kwong et al. 2014 ; Chen et al. 2019). Interestingly, aside from the Nanopore-only assembly, all assemblies recovered similar amounts of non-repeat regions. The PacBio-HiFi assembly showed a slightly larger non-repeat portion (Fig. 1b), likely due to improved recovery of duplicated genes, as indicated by a higher proportion of BUSCOs in the Complete-Duplicate category (Fig. 1a).

**Table 1.** Summary of genome assembly statistics for *Zancudomyces culisetae* using different sequencing technologies.

Sequencing method	Illumina	Illumina and Nanopore	Illumina and PacBio-CLR	Illumina and PacBio-CLR and Nanopore	Nanopore	PacBio-CLR	PacBio-HiFi
Assembler	SPAdes	SPAdes	SPAdes	SPAdes	Canu	Flye	HiFiASM &Finisher
Genome size (bp)	21,012,904	24,039,265	23,647,634	24,535,143	34,666,050	26,640,971	27,184,066
Coverage	78 x	N/A	N/A	N/A	844 x	468 x	48 x
Contigs No.	1,898	952	810	688	344	208	26
GC ratio (%)	35.67	35.47	35.46	35.42	35.28	35.58	35.56
Largest frg (bp)	367,220	1,383,685	1,061,931	1,065,925	3,086,553	1,691,663	5,239,357
N50 (bp)	37,998	128,795	126,571	232,805	322,842	525,018	2,524,970
L50	139	32	41	28	24	15	4
BUSCO % (complete [single-copy, duplicated], fragmented, missing)	70.1 [68.6, 1.5], 8.3, 21.6	71.3 [69.5, 1.8], 7.8, 20.9	71.3 [69.5, 1.8], 7.7, 21.0	71.6 [69.8, 1.8], 7.7, 20.7	63.7 [55.9, 7.8], 11.9, 24.4	71.7 [69.9, 1.8], 7.5, 20.8	71.9 [65.7, 6.2], 7.4, 20.7
Retroelements (bp)	414,226	1,888,948	1,470,941	2,056,224	6,723,120	3,836,832	3,031,068
DNA transposons (bp)	72,935	199,695	199,936	226,578	340,490	255,722	190,480
Rolling-circles (bp)	0	504	1,414	704	0	1,214	904
Unclassified (bp)	557,170	1,668,439	1,573,557	1,874,045	1,420,719	1,983,403	2,093,113
Small RNA (bp)	5,399	26,790	30,180	55,375	428,382	71,525	103,999
Satellites (bp)	0	0	5,720	2,626	0	0	0
Simple repeats (bp)	531,479	551,430	558,219	555,509	688,325	599,424	635,054
Low complexity (bp)	130,493	134,560	132,667	135,193	153,891	138,519	145,568
Total repeats (bp)	1,711,702	4,470,366	3,972,634	4,906,254	9,754,927	6,886,639	6,200,186
Non-repeat regions (bp)	19,301,202	19,568,899	19,675,000	19,628,889	24,911,123	19,754,332	20,983,880

The Nanopore assembly was an outlier, yielding the largest genome size across all assemblies, with the highest amounts of both non-repeat regions (119% to 129% of other assemblies) and repeat elements (1.4–5.7 × of other assemblies; Fig. 1b and Table 1). This inflation is likely attributable to sequencing errors inherent to Nanopore reads, which may have caused the assembler to incorrectly place redundant reads from the same genomic region in separate locations. Such misassemblies would artificially increase the total genome size (Fig. 2). In addition, the Nanopore assembly exhibited lower contiguity and a greater number of contigs (344), compared to the PacBio-CLR (208) and PacBio-HiFi (26) assemblies, further supporting this observation. Although all hybrid and long-read-based assemblies recovered more repetitive content than the Illumina-only assembly, the long-read only assemblies performed better, particularly in resolving retroelements and unclassified repeats (Fig. 1b and Table 1). This highlights the advantage of long-read sequencing for assembling complex genomic regions that are challenging to reconstruct using short-read data alone.

### High-quality genome assembly of *Z. culisetae*

**Fig. 3.**
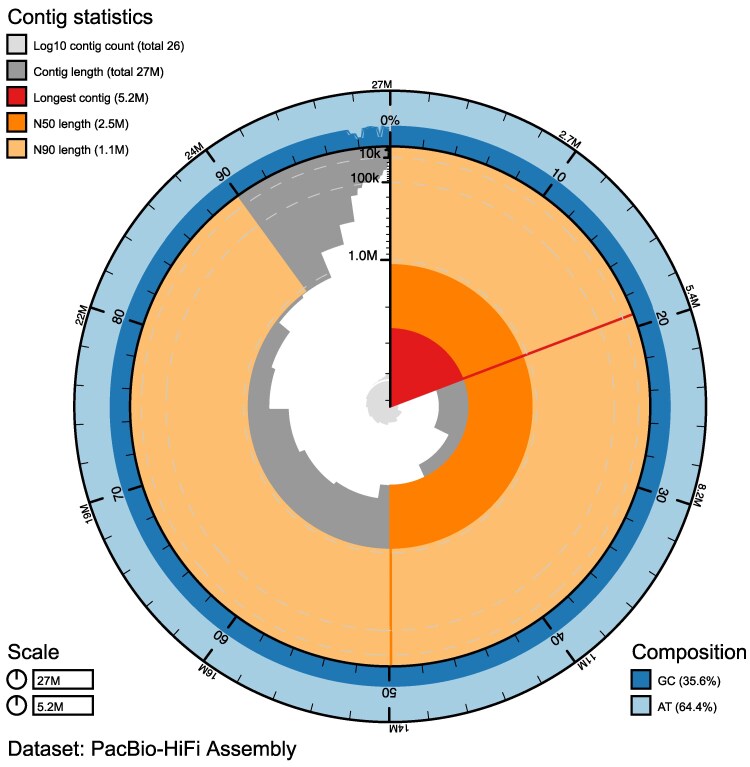
Snail plot of the best assembly for *Zancudomyces culisetae*, generated from PacBio-HiFi reads. The longest contig is highlighted in red (5.2 Mb), with remaining contigs arranged clockwise by size. Dark and light orange arcs indicate N50 and N90 values, respectively. The central gray spiral shows cumulative contig count on a log scale, with white lines marking each order of magnitude. The GC content is displayed as dark and light blue tracks.

The highest-quality *Z. culisetae* genome assembly was generated using PacBio HiFi reads, resulting in 26 contigs totaling 27.2 Mb, with an N50 of 2.5 Mb and a GC content of 35.56% (Fig. 3 and Table 1). The largest contig exceeds 5.2 Mb, and 11 contigs over 1 Mb together account for >93% of the genome (Fig. 3). These assembly metrics surpass those of all currently available genomes of insect gut-dwelling fungi, providing a high-quality reference for future research in this fungal lineage (Wang et al. 2018; Prakash and Wang 2024).

Although it represents the highest-quality assembly in this lineage, the *Z. culisetae* genome achieved a BUSCO completeness score of only 71.9% using the fungi_odb10 database. In addition to 7.4% Fragment content, ∼20.7% of conserved BUSCO genes were not detected in this insect gut-dwelling fungus. The fungi_odb10 database, primarily constructed from annotated genomes of Dikarya (Ascomycota and Basidiomycota), may introduce taxonomic bias that limits the detection of conserved genes in non-Dikarya taxa, such as *Z. culisetae* (Kickxellomycotina, Zoopagomycota). However, closely related free-living soil saprobes within Kickxellomycotina, such as *Coemansia reversa* (93.8%) and *Martensiomyces pterosporus* (95.1%), still exhibit high completeness scores (Chang et al. 2015; Amses et al. 2022; Wang et al. 2023). This contrast suggests that *Z. culisetae* has undergone substantial gene loss during its adaptation as an obligate insect gut symbiont, likely driven by prolonged exposure to host-derived food particles, enzymes, and metabolites produced by both the host and co-existing microbes (Frankel-Bricker et al. 2020; Reynolds et al. 2023). The observed gene reduction may be due to shared metabolic pathways with the hosts or co-existing microbes, a common feature observed in many endosymbionts (Pombert et al. 2012; Sloan and Moran 2012). This pattern of gene loss in insect gut-dwelling fungi has been consistently reported across multiple studies, regardless of sequencing methods (Wang et al. 2018, 2023; Prakash and Wang 2024). *Zancudomyces culisetae* and its close relatives have likely lost a core set of conserved genes through long-term co-evolution with their insect hosts, in contrast to their free-living relatives that share a most recent common ancestor (Chang et al. 2015; Wang et al. 2019, 2023; Amses et al. 2022).

### Genome annotation

The genome encodes 8,484 protein-coding genes, including 469 with alternative splicing, for a total of 8,953 predicted proteins distributed across 24 of the 26 contigs (excluding contig2 and contig19). InterProScan assigned functional annotation to 8,179 of the 8,953 proteins (91.35%), identifying 3,311 distinct Pfam domains, 4,362 proteins with predicted disordered regions, and 2,133 with coiled coil regions. Transcriptome reads mapped to the genome at a rate of 97.81%, further supporting the high completeness and accuracy of the assembly. The mitochondrial genome was identified using the MitoHiFi v2 pipeline and assembled as a linear sequence of 58,654 bp containing 60 genes, consistent with the previously reported *Z. culisetae* mitogenome (NC_006837.1) (Seif et al. 2005). The pipeline detected potential mitogenome on 2 contigs, contig2 and contig19, both of which lacked protein-coding genes based on earlier annotations. Reciprocal BLASTn analysis revealed that the contigs overlap by 61,188 bp (comparable to the mitogenome size) and share 99.90% sequence similarity. Accordingly, both contigs were designated as representing the mitogenome. An annotation graph for both contigs is included in the Supplementary materials (Supplementary File 1). Future efforts toward achieving a chromosome-level assembly will help resolve this redundancy.

### Genome biology and selenocysteine synthase in *Z. culisetae*

As one of the first microbial fungi cultured from mosquito larvae and a globally distributed fungal symbiont, *Z. culisetae* has been instrumental in foundational discoveries concerning the biology of insect gut-dwelling fungi. *Zancudomyces culisetae* has served as a model in numerous studies, including investigations of temperature and pH effects on fungal development, host specificity, responses to nutritional stress, and phylogenetic relationships (Horn and Lichtwardt 1981; Lichtwardt 1986; Horn 1989; Wang et al. 2013; Rooy et al. 2025). However, a major obstacle to advancing our understanding of their genome biology and the molecular mechanisms underlying interactions with mosquitoes has been the lack of high-quality genome assemblies. The genomic resource of *Z. culisetae* strain HAW-14–8 presented in this study addresses this gap and will serve as a model for genomic investigations aimed at uncovering the genome structure and hidden biology of this fungal symbiont. Notably, this strain was originally isolated from *Aedes albopictus*, an important vector of human infectious diseases, including yellow fever, dengue, and Chikungunya fever (Vega-Rúa et al. 2020; Bohers et al. 2024). The high-quality genome assembly of *Z. culisetae*, in the context of its innate association with a major disease vector, provides a critical foundation for exploring host–microbe interactions and the genetic basis of fungal symbiosis in the mosquito gut environment. These insights may ultimately support the development of biocontrol strategies for insect vectors.

The previously published *Z. culisetae* genome assembly (COL-18–3; ARSEF 9012), generated using Illumina short-reads, is highly fragmented, consisting of 1,954 scaffolds with an N50 of 26,472 bp and a BUSCO completeness score of 68.3% (Wang, White, Kvist, et al. 2016). It contains all essential Sec machinery genes and the SelenoH protein, features shared among Harpellales fungi, with the exception of selenocysteine synthase (SecS), whose absence was previously attributed to incomplete assembly (Wang, White, Kvist, et al. 2016; Mariotti et al. 2019). In the present study, we report a high-quality *Z. culisetae* genome assembled using PacBio HiFi long reads, in which a SecS homolog was successfully recovered, confirming that its absence in the earlier assembly was due to assembly limitations. Additionally, RNAseq data indicate that selenoproteins and their associated machinery genes are actively expressed, suggesting functional roles in the biology of these insect gut-dwelling fungi (Supplementary File 2).

### Conclusions and future research

Whole-genome sequencing has revolutionized genetic studies by providing unprecedented insights into genome architecture and full genetic landscapes of an organism (Goffeau et al. 1996; Gryganskyi et al. 2023). Illumina short-read sequencing has been widely utilized and particularly for species with reference genomes, including animals (Li et al. 2009; Gerdol et al. 2020), plants (Li et al. 2020; Guo et al. 2024), and fungi (Ahrendt et al. 2018; Lofgren et al. 2022). Its robust accuracy has been advantageous for detecting small variants and single nucleotide polymorphisms and thus been instrumental in genome-wide association studies and pangenome production across diverse organisms (Gerdol et al. 2020; Guo et al. 2024). However, the inherent short length of Illumina reads limits its ability to resolve repetitive elements and complex genomic regions, such as telomeric and centromeric repeats, transposable elements, and segmental duplications (Nagarajan and Pop 2013). These regions often exceed the read length, leading to ambiguous assemblies where repeats collapse into single consensus sequences. Third-generation sequencing technologies, such as Nanopore and PacBio have addressed these limitations by generating long-read solutions. PacBio High-Fidelity sequencing combines the benefits of long-read length and high accuracy by generating consensus reads from multiple passes of circularized DNA molecules, yielding accuracies >99.9% (Logsdon et al. 2020).

Our study highlights the advantages of PacBio HiFi sequencing for fungal genome assembly, offering a robust balance between sequencing accuracy, contiguity, and computational efficiency. While PacBio HiFi has higher per-base costs than Illumina, its ability to generate contiguous assemblies reduces the need for additional sequencing depth and complex downstream pipelines, ultimately offsetting costs. The high-quality *Z. culisetae* genome assembly provides a valuable resource for advancing research on insect gut fungi, addressing a critical gap in the field. In addition, the comprehensive comparison of sequencing technologies presented here establishes a framework for future genome projects in non-model fungal systems.

Future efforts should focus on achieving chromosome-level assemblies using ultra-long sequencing and chromatin conformation capture technologies. These advancements will further resolve complex genomic regions, including telomeres and provide deeper insights into the evolutionary and functional adaptations of *Z. culisetae* and related fungi. By establishing standardized guidelines and high-quality reference genomes, this study lays the foundation for future genomic research on non-model organisms, particularly those with specialized ecological niches.

## Supplementary Material

jkaf204_Supplementary_Data

## Data Availability

Sequence data and the final genome assembly for *Zancudomyces culisetae* HAW-14-8 (SAMN48099576) have been deposited in the NCBI database under BioProject accession PRJNA1254182. Predicted transcripts, proteins, and all assembly files used in comparative analyses are available at https://doi.org/10.5281/zenodo.15271765. Supplemental material available at G3 online.
