# Supplementary figures and images for: From fragmentation to resolution: high-fidelity genome assembly of Zancudomyces culisetae through comparative insights from PacBio, Nanopore, and Illumina sequencing
Source: G3 (Bethesda). 2025 Sep 1;15(11):jkaf204. doi: 10.1093/g3journal/jkaf204 (PMC12611240; doi:10.1093/g3journal/jkaf204)

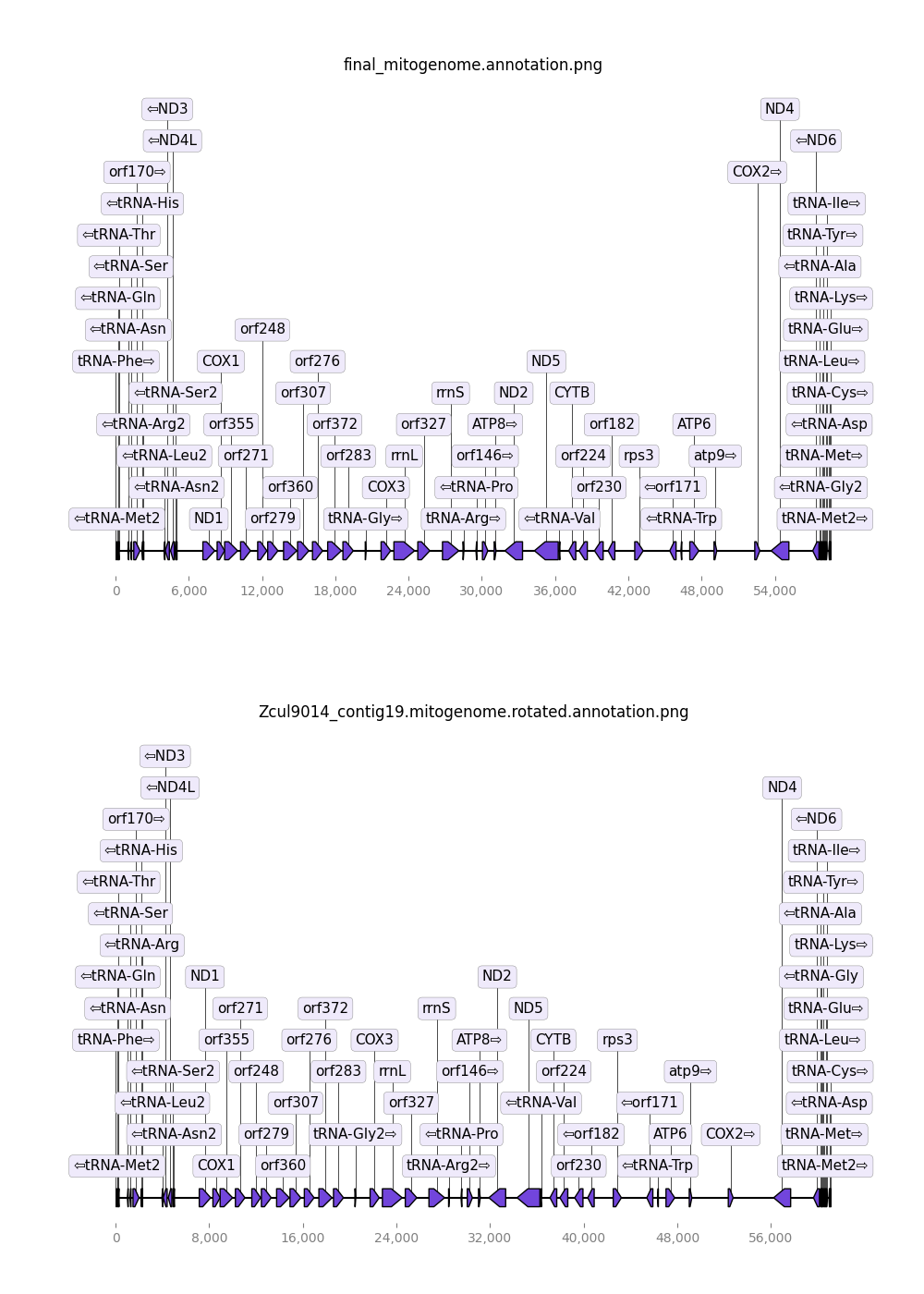

Supplement: jkaf204_Supplementary_Data [file jkaf204_supplementary_data.zip › Supplemental_File_1_G3-2025-406119.png]
